# Percutaneous cholangioscopy with biodegradable stent placement for stone disease on a background of a postanastomotic biliary stricture

**DOI:** 10.1055/a-2155-5203

**Published:** 2023-09-15

**Authors:** Apostolis Papaefthymiou, An Thang Ngo, George J. Webster

**Affiliations:** 1 Pancreaticobiliary Medicine Unit, University College London Hospitals (UCLH), London, United Kingdom; 2 Radiology Department, University College London Hospitals (UCLH), London, United Kingdom

Biliary access in patients with altered anatomy represents a limitation of endoscopic retrograde cholangiopancreatography (ERCP), with Roux-en-Y reconstruction being one of the most challenging postoperative variants 1 . In the setting of benign stricturing, the use of plastic or metal stents may be precluded by difficult access for their removal. Percutaneous cholangioscopy combined with biodegradable stents may provide a solution 2 .

A 64-year-old woman with a history of a choledochal cyst treated with bile duct resection and hepaticojejunostomy 25 years previously was referred with recurrent cholangitis due to an anastomotic stricture and large intrahepatic stones. Previous anastomotic stricture dilation had not prevented further stone formation, and surgical revision was not considered an optimal option. Percutaneous transhepatic drainage (PTD) was performed, with an 11-Fr sheath placed, which was followed by cholangioscopy (Spyglass Discover; Boston Scientific Inc.) ( Video 1 ). A significant number of stones were fragmented with electrohydraulic lithotripsy (EHL), but effective clearance of these fragments from the duct could not be achieved because of the stricture.

**Video 1** Percutaneous cholangioscopy with placement of a biodegradable stent for stone disease on a background of a postanastomotic biliary stricture.


Remodeling of the anastomotic stricture was attempted using an endoscopic balloon-expandable biodegradable stent system (UNITY-B; AMG International GmbH). A cholangiogram confirmed the presence of residual stone burden above the anastomotic stricture ( Fig. 1 ). An extraction balloon advanced over a guidewire across the anastomotic stricture failed to clear the biliary tree of stone debris, owing to difficulty pushing the stones and the balloon (8.5–11.5 mm) through the stricture. The balloon was therefore removed and the stent delivery system was passed over the 0.035-inch guidewire, with the aim of remodeling the anastomotic stricture. The stent position was confirmed radiographically based on fluoroscopic identification of the radiopaque markers at the proximal and distal ends of the stent, and the biodegradable stent (10 × 57 mm) was deployed by inflating the incorporated inflation balloon. A follow-up cholangiogram 6 weeks after the procedure confirmed resolution of the stricture and clearance of the debris ( Fig. 2 ).

**Fig. 1:**
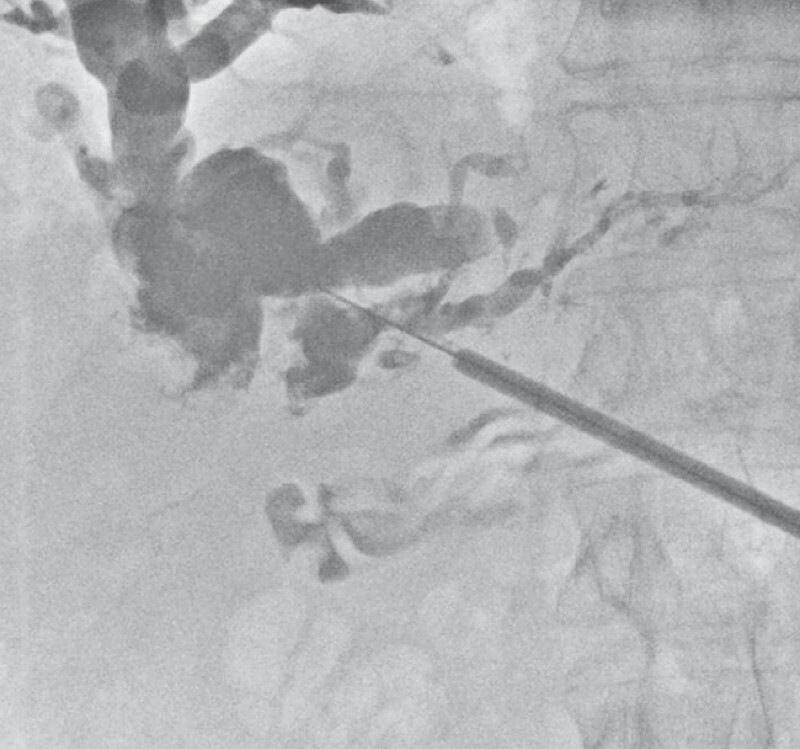
Initial cholangiogram showing the stone burden above the anastomotic stricture.

**Fig. 2:**
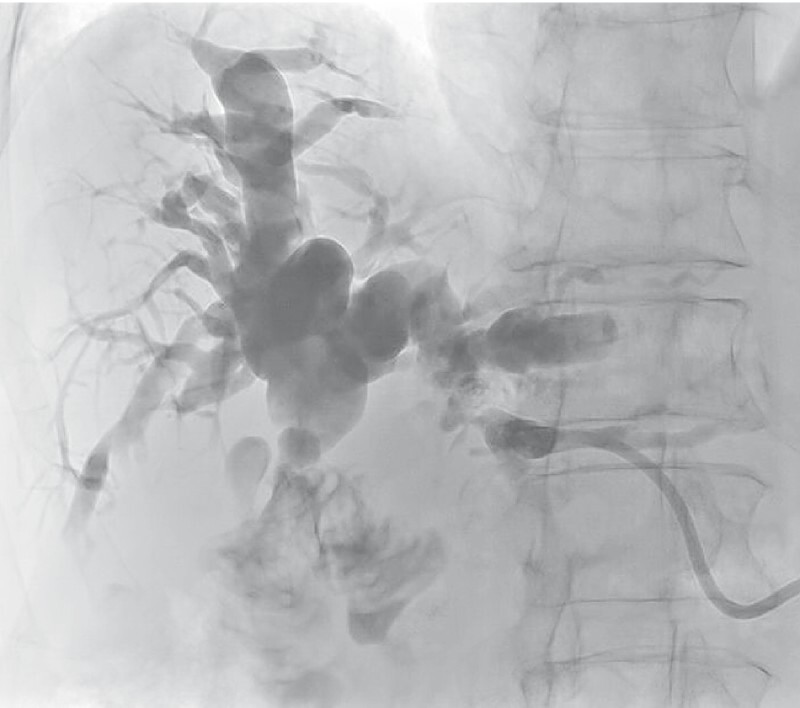
Follow-up cholangiogram 6 weeks after deployment of the biodegradable stent showing stricture resolution and clearance of the debris.

Percutaneous cholangioscopy combined with biodegradable stent placement represents a novel approach in the management of stones above biliary strictures in patients with surgically altered anatomy.

Endoscopy_UCTN_Code_TTT_1AR_2AG

## Notes

Endoscopy E-Videos

https://eref.thieme.de/e-videos
*E-Videos* is an open access online section of the journal *Endoscopy* , reporting on interesting cases and new techniques in gastroenterological endoscopy. All papers include a high-quality video and are published with a Creative Commons CC-BY license. Endoscopy E-Videos qualify for HINARI discounts and waivers and eligibility is automatically checked during the submission process. We grant 100% waivers to articles whose corresponding authors are based in Group A countries and 50% waivers to those who are based in Group B countries as classified by Research4Life (see: https://www.research4life.org/access/eligibility/ ). This section has its own submission website at https://mc.manuscriptcentral.com/e-videos
